# Current Metabolic Engineering Strategies for Photosynthetic Bioproduction in Cyanobacteria

**DOI:** 10.3390/microorganisms11020455

**Published:** 2023-02-11

**Authors:** Alessandro Satta, Lygie Esquirol, Birgitta E. Ebert

**Affiliations:** 1 Australian Institute for Bioengineering and Nanotechnology, The University of Queensland, St Lucia, QLD 4072, Australia; 2 Department of Biology, University of Padua, 35100 Padua, Italy; 3 Centre for Cell Factories and Biopolymers, Griffith Institute for Drug Discovery, Griffith University, Natha, QLD 4111, Australia

**Keywords:** methylerythritol phosphate (MEP) pathway, photosynthesis, isoprenoids, lipids, biofuels, gene editing, CRISPR, riboswitch, ribo-regulators, nano-compartments

## Abstract

Cyanobacteria are photosynthetic microorganisms capable of using solar energy to convert CO_2_ and H_2_O into O_2_ and energy-rich organic compounds, thus enabling sustainable production of a wide range of bio-products. More and more strains of cyanobacteria are identified that show great promise as cell platforms for the generation of bioproducts. However, strain development is still required to optimize their biosynthesis and increase titers for industrial applications. This review describes the most well-known, newest and most promising strains available to the community and gives an overview of current cyanobacterial biotechnology and the latest innovative strategies used for engineering cyanobacteria. We summarize advanced synthetic biology tools for modulating gene expression and their use in metabolic pathway engineering to increase the production of value-added compounds, such as terpenoids, fatty acids and sugars, to provide a go-to source for scientists starting research in cyanobacterial metabolic engineering.

## 1. Introduction

Cyanobacteria are photosynthetic unicellular microorganisms with powerful biotechnological features. They are Gram-negative prokaryotes that belong to the bacterial domain and are considered one of the oldest and largest groups of bacteria on Earth. The oldest fossil dates back to the Archean era. Cyanobacteria were essential for forming the biosphere, creating oxygenating conditions by releasing O_2_ into the atmosphere [1]. They are also able to fix nitrogen, occupying a prominent role in the nitrogen cycle [2]. Given their long history, their ability to adapt to environmental changes on Earth is one of their principal characteristics. For instance, they have differentiated specialized nitrogen-fixing cell types, facilitating the dispersion of species [3] and are found in marine, freshwater and terrestrial environments. Cyanobacteria possess a typical prokaryotic cellular organization; however, they lack the cell wall usually found in bacteria. Plant chloroplasts are thought to be derived from endosymbiotic cyanobacteria [4], explaining the similarity of their photosynthetic apparatus embedded in the thylakoid membranes [5].

Despite being a relatively ‘young’ organism from a biotechnological point of view (compared to the well-characterized industrial chassis *Escherichia coli* and *Saccharomyces cerevisiae*), cyanobacteria’s capability to use solar energy for generating reducing power and energy along with their prokaryotic cellular organization make them attractive biotechnological agents to produce valuable compounds. Cyanobacteria convert inorganic carbon dioxide (CO_2_) and H_2_O into biomass and valuable products and some species can also fix molecular nitrogen. They have a clear advantage compared to the microorganisms, such as *E. coli* and *S. cerevisiae*, which are currently the preferred cell platforms in industrial biotechnology, but rely on reduced carbon and nitrogen sources, typically sugars and ammonia, increasing the production costs of the target compounds [6,7]. Their photosynthetic biomass production rate is also higher than that of plants [8,9,10]. Moreover, genetic modifications of cyanobacteria are faster and more efficient than in plants or algae [8,9]. Many molecules of commercial interest are natively produced by cyanobacteria, for instance: terpenoids, chlorophylls, fatty acids, sugar and amino acids [11,12]. Together, these traits make cyanobacteria ideal candidates for sustainable, low-cost biological production of high-value chemicals.

Various strategies have been implemented to optimize cyanobacteria for bioproduction, including flux enhancement through a determined pathway, removal of competitive pathways, or augmenting cell fitness against high product concentrations [13,14,15,16]. Most of the metabolic engineering approaches have led to scarce results in terms of productivity and titer in comparison to those achieved in heterotrophic microbes [10]. The lack of knowledge about cyanobacterial regulatory mechanisms, a gap in available genetic tools and the inconsistency in performance of characterized genomic parts across different cyanobacterial strains can explain this discrepancy. The increased number of cyanobacterial genome sequencing data (3858 cyanobacterial genome assemblies available in GenBank [17]) has greatly facilitated the integration of transcriptomics, proteomics and metabolomics studies and helped raise awareness beyond model organisms and identify new industrial relevant species [18]. The evolution of the synthetic biology paradigm as the systematic reconstitution of new standardized biological parts, modules and devices to produce a particular cellular output has further spurred the availability of well-characterized genetic parts (e.g., promoters, ribosome binding sites (RBS) and coding sequences) and improved methods for cyanobacteria metabolic engineering.

This review will introduce the principal cyanobacterial model organisms and lesser-known species with relevant biotechnological traits and applications. Secondly, we will focus on available genetic engineering and synthetic biology tools. Finally, we present the main achievements in terms of metabolic engineering of cyanobacteria, taking a closer look at the production of terpenoids, fatty acids and carbohydrates. We will also introduce several new engineering approaches still majorly unexplored in cyanobacteria and highlight some challenges and prospects of cyanobacterial cell factory development.

## 2. Model Organisms and Emerging Species

Due to the diverse ecological niches colonized by cyanobacteria, many studies focus on species with unique physiological features and have identified promising photosynthetic cell chassis candidates with industrially relevant traits. The main requirements for enabling metabolic engineering of an organism are the availability of an annotated genome sequence, amenability to genetic manipulation and rapid and efficient methods for generating mutants. Several cyanobacteria species fulfill these requirements (e.g., *Synechocystis* sp. PCC 6803, *Synechococcus* sp. PCC 7002, *Synechococcus elongatus* sp. PCC 7942 and *Anabaena* sp. PCC 7120) and have become model organisms in cyanobacteria physiology and metabolic engineering studies.

The freshwater cyanobacterium *Synechocystis* sp. PCC 6803 (Syn6803) is one of the most widespread cyanobacteria. This phototrophic organism was isolated in 1968 in Oakland, California [19]. Because of biochemical similarities with the chloroplast of green plants, it was used as a model for stress response and adaptation in higher plants [20] and is very well studied and understood. This is reflected by the 9380 scientific articles referring to this cyanobacterial strain [21]. Its genome was also the first completely sequenced cyanobacterial genome [22,23]. The strain possesses great potential for biotechnological applications, primarily because of its versatile carbon metabolism, compared to other photosynthetic organisms, and broad generation of valuable compounds. The transformation efficiency, genomic accessibility, the increasing number of synthetic biology tools available for genome editing and the ability to grow photo-heterotrophically at the expense of glucose make Syn6803 the most popular cyanobacterial model organism. More information about this organism can be found in the review from Yu et al. [24] and Table 1.

*Synechococcus elongatus* sp. PCC 7942 (Syn7942) (former *Anacystis nidulans* R2) is an obligate photoautotrophic freshwater cyanobacterium isolated for the first time in the San Francisco Bay area of California in the mid-1980s [25]. It was the first cyanobacterium transformed with exogenous genetic material [26]. Its physiology has been extensively studied, including carbon and nitrogen assimilation and regulation, and its adaptation and fitness under various environmental stresses has been investigated [27]. Syn7942 has been considered the main organism for exploring the prokaryotic circadian clock system [28]. Syn7942 is genetically tractable as it possesses a minimal genome (Table 1) [29] and a noticeable number of synthetic biology tools are available for modulating its protein expression. Thus, Syn7942 has been extensively used as a cell chassis for heterologous production of a plethora of compounds [30,31,32,33,34], as summarized in Table 1 and [25,35,36]. 

*Synechococcus elongatus* sp. PCC 7002 (Syn7002) is a unicellular marine cyanobacterium, first isolated on the Magueyes Island of Puerto Rico in 1963 [37]. Syn7002 has a remarkable halophilic and high-light tolerance [38] and is considered one of the fast-growing cyanobacteria (Table 1) [39]. Under high-light conditions, Syn7002 reduces antenna size and redistributes carbon flows to fuel biomass formation while increasing cell volume on average by about 3-fold [39]. Together with established techniques for protein overproduction and a wide range of synthetic biology tools available [40], it is a compelling candidate for large-scale bioreactor cultivation, its only drawback being its obligate requirement for exogenous vitamin B_12_ (cobalamin); for further details, see Table 1 and [36,41]. 

*Anabaena* sp. PCC 7120 (Ana7120), formerly known as *Nostoc muscorum*, is a filamentous, non-branching, autotrophic, freshwater cyanobacterium [42,43]. It was first isolated in 1971 from the algal collection at Iowa State University [44]. It is characterized by differently colored cells, grows aerobically under various temperature and light intensity regimes and can fix atmospheric nitrogen through its nitrogenase complex. Under nitrogen deficiency, Ana7120 differentiates into non-photosynthetic cells called heterocysts, specialized in nitrogen fixation [45]. Ana7120 is therefore considered the model organism for studying heterocyst differentiation and multicellularity. Ana7120 also attracted attention for its native production of industrial-relevant secondary metabolites such as polyketides and the sesquiterpenoid geosmin [46]. Its genome has been completely sequenced and it can easily be manipulated with an array of genetic tools for gene expression control, genome editing and reporter fusions [47,48]; further details are given in Table 1 and [49,50].

New industrially relevant organisms—besides those four model organisms, some fast-growing cyanobacteria strains were recently discovered that overcome the disadvantages of Syn6803 and Syn7942 in terms of low productivity, slow growth, inability to grow at elevated temperatures and lack of stress tolerance. 

*Synechococcus elongatus* UTEX 2973 (Syn2973) is closely related to Syn7942 as their genomes differ by only 55 single nucleotides [51]. This recently discovered strain is characterized by thermotolerance and can grow twice as fast as Syn7942 and Syn6803 (Table 1). However, in contrast to these model strains, it is not naturally transformable [51]. Despite its recent discovery, several proteomic, transcriptomic and metabolomic studies have already been conducted with this strain [52,53,54]. Under nitrogen-depleted conditions, Syn2973 can accumulate glycogen to over 50% of dry cell weight [55] and has been considered a valuable feedstock for yeast-based biofuel production [56]. Some of the most recent synthetic biology techniques were already successfully applied in Syn2973, greatly facilitating rational engineering and making it one of the most promising candidates to be developed into an efficient photosynthetic chassis [57].

The cyanobacterium *Synechococcus elongatus* sp. PCC 11801 (Syn11801), isolated in 2018 from water samples collected from Powai Lake, Mumbai, India [58], shares 83% homology with Syn7942. It displays ultra-fast growth at elevated temperatures and light intensity (Table 1). Further promising biotechnological attributes are its amenability for natural transformation and cultivation with seawater. Syn11801 was efficiently engineered to produce succinic acid, confirming its biotechnological potential [59]. Along Syn11801, other less characterized fast-growing strains displayed interesting biotechnological features, such as *Synechococcus elongatus* sp. PCC 11802 (Syn11802). This strain grows even faster than Syn11801 under unlimited growth conditions (Table 1). It has outstanding carbon fixation capabilities, probably because the Calvin cycle key enzymes are not repressed under elevated CO_2_ concentrations [60]. Excellent CO_2_ fixation performance is a key biotechnological feature for enhancing chemical production. Another fast-growing cyanobacterium is *Synechococcus* sp. PCC 11901 (Syn11901) (Table 1). This euryhaline organism can tolerate a wide range of salinities, which is economically advantageous because it can potentially avoid using freshwater and can decrease contamination risks. Moreover, engineered strains of Syn11901 showed the highest capacity of free fatty acid production reported in cyanobacteria [61].

Some less-known genera of cyanobacteria have attracted attention for their ability to produce structurally complex compounds. These strains are being screened for new metabolites with unusual bioactivities, in particular, cyanobacteria belonging to the genera Nostoc, Lyngbya and Microcystis, which are studied for the production of antivirals (e.g., Nostoflan), the UV-screening compound Scytonemin, anticancer compounds (e.g., Apratoxin A and Curacin A) and enzyme inhibitors (e.g., Micropeptins) [62].

Cyanobacteria, such as the filamentous Spirulina, have also been used for single-cell protein (SCP) production. For instance, the dried biomass of the oxygenic photosynthetic bacterium *Arthrospira platensis* is used as a protein and vitamin source in human diet supplements [63]. It was first discovered in 1844 near Montevideo, Uruguay [64] and is the only cyanobacterium listed as a generally recognized as safe (GRAS) organism by the U.S. Food and Drug Administration [65]. This cyanobacterium has also been investigated to elucidate the origin and biochemical and biophysics mechanisms of oxygenic photosynthesis. The first reported genomic modification of *A. plantensis* C1 has been performed by transposon mutagenesis (Table 1) [66]. Recently, Jester et al. engineered *A. plantensis*, by natural transformation, for stable, high-level expression of bioactive antibodies against campylobacter infections (Table 1) [67]. Furthermore, *A. plantensis* was engineered for the production of the pain reliever Acetaminophen (Table 1) [68]. These new rational engineering strategies may pave the way for future orally-delivered *A. plantensis* strains as human therapeutics.

*Fremyella diplosiphon* is a freshwater cyanobacterium isolated from a Connecticut lake in 1952, which possesses one of the largest genomes amongst bacteria (Table 1). It has attracted attention because of its exceptional chromatic acclimation capabilities. In this process, *F. diplosiphon* changes its pigment composition to maximally absorb the available photons of light to support photosynthesis [69]. It is equipped with one of the most intricate light-sensing systems composed of 27 different phytochrome superfamily members for light color sensing [70]. It has, therefore, become the model cyanobacterium organism for studying complementary chromatic adaptation. 

*Prochlorococcus* is the smallest (spherical diameter from 0.5 to 0.7 μm) and most abundant photosynthetic organism in the ocean [71]. It was first isolated in 1988 from the bottom of the euphotic zone in the Sargasso Sea [72]. Distributed all over the Earth, it can be grouped into different ecotypes based on the habitats it colonizes [73]. Because of its abundance and adaptability, it has become a model for studying biological diversity across diverse scales, achieved by integrating multi-omics studies and global ecosystem modeling (Table 1) [74]. Moreover, *Prochlorococcus* possesses some unique features amongst cyanobacteria, such as a photosynthetic apparatus composed of the divinyl derivatives of chlorophyll a and b (Chl-a_2_ and Chl-b_2_), a photosystem closer to that present in green plants and algae [73] and the ability to generate the volatile hemiterpenoid isoprene [75]. Although it has attractive biotechnological features, it is recalcitrant to DNA transfer and to growing axenically on solid media [76,77].

*Gloeobacter violaceus* is a rod-shaped primordial cyanobacterium isolated for the first time from limestone rocks in Kernwald, Switzerland in 1974 [78]. It lacks the thylakoid membrane and instead shuttles protons from the cytoplasm to the periplasm. Its archaic photosystem is solely composed of menaquinone and it does not possess the common cyanobacterial phylloquinone [79]. It is thought to be the first cyanobacterium and is the object of studies on the evolution of cyanobacteria lineage and the development of anoxygenic photosynthesis [80]. The main characteristics are summarized in Table 1.

*Thermosynechococcus elongatus* is a thermophilic rod-shaped cyanobacterium with an optimal temperature of 55 °C isolated from a hot spring in Beppu, Kyushu, Japan, in 1978 [81]. It is capable of natural transformation [82] and its genome has been fully sequenced (Table 1) [83]. Because of the thermostability of the proteins of its photosynthetic complex, it has become the model cyanobacterium organism for studying the biophysics of photosynthesis and the structure of the photosystem. Several crystal structures of many components of this intricate system have been solved [83,84,85,86,87].

**Table 1:** Main features of cyanobacteria strains.

Organism	Genome Size (Mbp)	Plasmids	Doubling Time (hours) *	Growth Temperature *	CO_2_ Partial Pressure (%) *	Light Intensity (µmole photons m^−2^ s^−1^) *	Transformation Methods	Metabolism	Features	References
Syn6803	3.5	4	5.1	35 °C	5	790	NT ****, Conjugation, Electroporation	Mixotrophic, Autotrophic	Model organism, best studied cyanobacterium, numerous synthetic biology tools available	[22,24,88]
Syn7942	2.69	1	4.1	38 °C	3	300	NT ****, Conjugation, Electroporation	Autotrophic	Model organism, especially suited for circadian clock studies	[26,28,35]
Syn7002	3.0	7	2.5 2.5 **	30 °C 3 °C **	2 1 *	760 250 **	NT ****, Conjugation, Electroporation	Mixotrophic, Autotrophic	Model organisms, high cell doubling rate; resistant to salt and high light conditions	[39,89]
Ana7120	7.2	6	18–24	23–30 °C	2	160	Conjugation	Mixotrophic, Autotrophic	Model organism, nitrogen-fixing strain	[90,91,92]
Syn2973	2.69	2	2.1	41 °C	3	500	Conjugation, Electroporation	Autotrophic	Highest growth rate, numerous synthetic biology tools available	[51]
Syn11801	2.7	/	2.3	41 °C	0.4	1000	NT ****, Conjugation, Electroporation	Autotrophic	Fast-growing strain, thermophil	[58,59]
Syn11802	2.7	/	2.8	38 °C	1	1000	NT ****, Conjugation, Electroporation	Autotrophic	Fast-growing strain, thermophil	[60]
Syn11901	3.0	1	2.1	38 °C	1	660	NT ****, Conjugation	Mixotrophic, Autotrophic	Fast-growing strain, resistant to high-stress conditions	[61,93]
*A. platensis* C1	6.62 ***	/	~60	30 °C	0.3	108	NT ****, Electroporation	Mixotrophic, Autotrophic	GRAS organism, production of proteins and pigments	[66,67,68,94,95]
*F. diplosiphon*	9.9 ***	14	~20	28–30 °C	N/A	30	Conjugation, Electroporation	Autotrophic	Suited for studying light absorption and photomorphogenesis	[96,97,98,99,100]
*P. marinus*	1.6	/	~24	20 °C	N/A	100	/	Autotrophic	Suited as biomarker for ocean metabolism	[77,101,102]
*G. violaceus* 7421	4.6	/	~73	25 °C	N/A	5	Conjugation	Autotrophic	Suited for studying anoxygenic photosynthesis	[103,104,105,106]
*T. elongatus* BP-1	2.6	/	6	55 °C	1.5	200	Electroporation	Autotrophic	Thermophilic cyanobacterium, ideal for studying cyanobacterial photosystem	[83,107,108]

* CO_2_%, temperature and light intensity correspond to the parameters used to achieve the fastest cell doubling rate for each cyanobacterial strain. ** Nitrogen-depleted conditions. *** Draft Genome Sequence. **** NT, natural transformation.

## 3. Current Methods for DNA Transfer into Cyanobacteria

Transferring heterologous or mutated genes through transformation is essential for rational engineering approaches to exploit cyanobacteria as a recombinant production system. Several methods exist and are described in the following.

### 3.1. Genomic Integration and Extrachromosomal DNA Insertion

Efficient transfer of exogenous DNA molecules into cyanobacteria can be achieved using “suicide” vectors for chromosomal gene transfer. This involves integrating the DNA fragment into cyanobacterial chromosomes by double homologous recombination and plasmid transformation using self-replicating plasmids [109]. Chromosome integration may be used to characterize endogenous genes using classic loss of function studies, where an antibiotic resistance cassette replaces the endogenous gene. For heterologous gene expression, genomic neutral sites are often targeted [110], which can be disrupted with no effect on cell metabolism and physiology [111,112] and allow stable expression of the introduced gene [111].

For maintaining a stable mutant genotype, the mutation must be present in all chromosomal copies to be retained stably and not be rejected [110]. This condition of complete genome segregation is time-consuming because most cyanobacteria are polyploid organisms with multiple chromosome copies, up to 53 in Syn6803, depending on growth phase and environmental parameters [111,113]. The individuation of positive mutants is achieved by antibiotic resistance selection. To enforce the mutation of all chromosomes, single positive colonies are sequentially re-streaked on a medium containing increasing antibiotic concentrations [114]. This process can take up to two months, depending on the strain used. Recently, Pope et al. showed that much quicker gene segregation could be achieved by reducing the phosphate concentration in the growth medium because low phosphate concentration increases the proportion of monoploid cells. However, this was accompanied by a 100-fold drop in transformation efficiency [115]. Analogously, Riaz et al. found out that the reduction of phosphate and agar concentration in the growth medium and high temperature are key parameters for reducing polyploidy and accelerating segregation process [116].

Self-replicating plasmids can alternatively be used for heterologous gene expression to avoid long segregation times. Shuttle vectors that replicate both in *E. coli* and cyanobacteria facilitate plasmid cloning and maintenance [117]. However, the major disadvantage of their use is the narrow host range in which they are available. Due to species-specific replicon systems, usability in different cyanobacterial species is complicated [118,119]. This drawback has been addressed by creating RSF1010-based plasmids with broader inter-species compatibility [117]. The popular cyanobacterial RSF1010 shuttle vector is the pPMQAK1 plasmid, which is supposed to replicate most consistently. However, the low copy number (on average 10–20 copies per *E. coli* cell) compromises its use, as transformation methods require high amounts of plasmid (3–4 µg) [119]. Several variants have been developed that show better transmissibility [120], are streamlined to follow the Standard European Vector Architecture (EVA) structure [121] and facilitate modular cloning [122].

Recent studies on the native plasmid replication systems of Syn6803 (Table 1) revealed the presence of two genetic elements (*Slr7037* and *ssr7036*), in the plasmid pSYSA, necessary for plasmid replication and extrachromosomal maintenance [123]. The insertion of *Slr7037* (encoding for a cyanobacterial replication initiator protein, CyRepA1) and *ssr7036* (encoding for a RNase E-mediated cleavage protein) into pUC19 backbone, which is not functional in cyanobacteria, enabled independent replication in Syn6803 and Syn7942 [123]. Thus, it may serve as a shuttle vector between cyanobacteria and *E. coli*.

### 3.2. Transformation Methods

The transformation of cyanobacteria and its methodology have been intensely investigated [26,124,125,126,127]. Three main transformation methods are available for cyanobacteria: natural transformation, conjugation and electroporation (Table 1).

#### 3.2.1. Conjugation

Conjugation is a widespread and often preferred method for transferring DNA into cyanobacteria [109] because of its high efficiency and broad applicability amongst different species [125]. In triparental mating, two different *E. coli* strains are used: a helper strain harboring a conjugative plasmid (carrying the *mob* genes necessary for mobilizing the genetic elements) and a donor strain carrying the cargo plasmid containing an origin of replication (*oriT*), the sequence necessary for conjugation, and the gene(s) of interest to be transferred into the recipient cyanobacterial cells. Some species require additional vectors for optimizing conjugation efficiency. For instance, Ana7120 is co-transformed with the helper plasmid pRL623, which encodes three methylases to prevent the digestion of the mobilizer plasmid [128].

#### 3.2.2. Electroporation

Electroporation involves applying a high-intensity electrical field to generate pores in the cell membrane, allowing DNA entry. So far, electroporation has been established for only a few cyanobacterial species, including *A. platensis*, *Anabaena* sp., *Synechococcus* sp., *Synechocystis* sp., *F. diplosiphon* and the nitrogen-fixing *Plectonema boryanum* [68,127]. Optimal voltage and pulse duration for high-efficiency transformation are species-specific [129]. Besides plasmid transfer, electroporation has also been successfully used to transform Syn6803 and Syn7942 with linear PCR fragments [130,131,132]. Using linear DNA fragments instead of plasmids is advantageous as it reduces laborious cloning steps and, therefore, costs and time but is prone to nuclease degradation of the fragments [133]. Recently, the efficiency of inserting linear PCR fragments into Syn7942 was improved by the addition of EDTA, which inhibits DNase activity [130].

#### 3.2.3. Natural Transformation

Natural transformation describes the uptake and maintenance of exogenous DNA by bacterial cells. This process is the basis of horizontal gene transfer between prokaryotic organisms, one of the leading forces contributing to the evolution of the prokaryotic domain [134]. In cyanobacteria, the retractile pilus T4P makes contact with the extracellular DNA and its retraction pulls the foreign DNA inside the cell. Several cyanobacteria are known to be naturally transformable such as the model organisms Syn6803, Syn7942 and Syn7002 [26,135,136]; the thermophilic *T. elongatus* sp. PCC BP-1 [82], the fast-growing strains Syn11801, Syn11802 and Syn11901 [58,60,61], the bloom-forming cyanobacterium *Microcystis aeruginosa* sp. PCC 7806 [137] and the filamentous *Phormidium lacuna* and *A. platensis* [67,68,138]. It is also possible to enable natural transformation through genetic engineering. The introduction of the *pilN* gene from Syn7942 into Syn2973 led to the acquisition of natural transformation competency, although with lower efficiency than Syn7942 [139]. The uptake efficiency relies on the concentration and length of the DNA, the cyanobacterial strain used and the growth phase [129]. Syn7002′s and Syn6803′s competency drastically decreases from exponential to stationary phase [140]. The circadian clock also seems to play a role in cyanobacterial transformation efficiency; the pili genes are overexpressed in darkness or low light conditions. Accordingly, the competency of cyanobacteria cells dramatically increases during darkness [140].

## 4. Tools for Manipulating Gene Expression in Cyanobacteria

The generation of predictable cellular output from utilizing standardized biological parts stands at the base of synthetic biology. Availability and reproducible behavior of well-characterized parts are essential for building functional genetic circuits and efficient microbial engineering. Most synthetic biology designs, fully operative in heterotrophic organisms such as *E. coli*, cannot be translated into cyanobacteria. For example, promoters and RBS that strongly modulate gene expression in *E. coli* do not perform well in cyanobacteria [141]. To date, the number of molecular tools for engineering cyanobacteria is far from that developed for *E. coli* and *S. cerevisiae*, but significant progress has been made in expanding and improving the cyanobacterial toolbox.

### 4.1. Increasing Gene Expression by Promoter Engineering

The most common way to overexpress genes in cyanobacteria is to use strong constitutive native promoters, but only a few have been described to date. In this category, the most common promoters control the expression of genes encoding components of the photosynthetic apparatus and are often light-regulated. Their application will be described in the following section.

#### 4.1.1. Strong Native Promoters

Amongst the endogenous promoters controlling the expression of the genes of the photosynthetic apparatus, P*_psbA2_* is a light-regulated promoter that natively drives the expression of the D1 protein of the photosystem II apparatus in Syn6803 [142]. It has been used for the light-inducible, heterologous expression of an isoprene synthase gene in Syn6803 [143]. P*_cpcB_* is another strong constitutive promoter that controls the expression of the C-phycocyanin operon (*cpc*), which was used to engineer Syn6803 for the heterologous production of ketone bodies [144]. Different cyanobacterial variants of the constitutive promoter P*_rbcL_*, which natively powers the expression of ribulose 1,5-diphosphate carboxylase/oxygenase (RuBisCO), are also frequently used [40,145,146].

The drawbacks of using strong native promoters are that strong gene expression is often limited to specific cyanobacteria species and that expression strength often oscillates with the circadian rhythm [147]. One example is P*_rbcL_* from Ana7120; the promoter was successfully deployed to drive fatty acids and ethanol production in Syn6803 [148,149] but showed a lower expression strength than P*_sbaA2_* and the heterologous, inducible promoter P*_trc1O_*. Similarly, it displayed a lower activity in Syn7002 compared to its native strong promoters, P*_A2520_* and P*_A2579_* [40].

#### 4.1.2. Synthetic Inducible Promoters

Synthetic promoters lead to increased and consistent expression strength, as they function orthogonally to the cell’s native regulatory network. While not entirely synthetic, the systematic modification of native promoters has been central to creating new variants with improved activity profiles. By manipulating the consensus sequence of core promoter elements, Wegelius et al. created a minimal synthetic promoter for heterocyst-specific expression in *Nostoc punctiforme* ATCC 29133, resulting in a 10-fold higher reporter expression [150]. Truncation of the P*_cpcB_* promoter and the addition of multiple transcription factor binding sites led to the very strong Syn6803 promoter P*_cpc560_* [151]. Similarly, gene expression driven by a truncated version of P*_psbA2_* displayed a 4-fold increment compared to the wild type [152]. Synthetic promoters have also been created to function in diverse cyanobacteria. Promoters of the series J23 [153] were shown to perform strongly in Ana120, Syn6803, Syn7002, Syn7942 and Syn2973 and synthetic promoter libraries recently created by mutation of the strong promoters P*_cpcB_* and P*_rbcL_* from Syn7942 are applicable in Syn8001 and Syn8002 [47,48,139,154,155].

#### 4.1.3. Chemically Inducible Promoters

**Figure 1:**
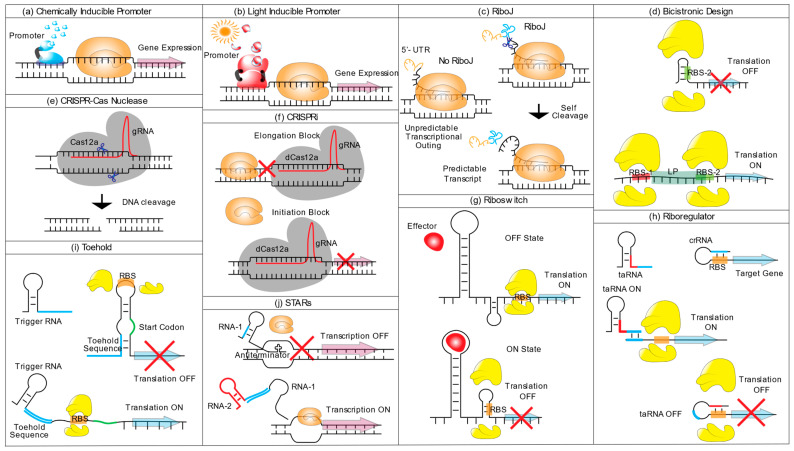
Schematic representation of synthetic biology tools available for cyanobacteria. (**a**) Chemically inducible promoter, upon binding of a small chemical inducer (blue shapes), the activator binds to the promoter leading to transcription activation (violet arrow) through the activity of RNA polymerase II (orange shape). At the same time, a repressor dissociates from the DNA upon inducer binding (not shown). (**b**) Light inducible promoter, light-sensing proteins (red shapes) are (de)activated upon light induction (red sun) and induce a cascade that results in transcription activation (grey arrow) or repression. (**c**) RiboJ, the self-cleavage activity of RiboJ (blue line), cleaves the variable 5′-UTR sequence upstream of the ribozyme (orange line), leaving a shorter mRNA (black) with standardized 5′-UTR (**d**) Bicistronic design; to avoid translation obstruction through RNA secondary structures of the RBS (RBS-2) (upper figure), a short leader peptide (LP) sequence with its own RBS (RBS-1) is placed upstream of the GOI. During translation of LP, the helicase activity of the ribosome resolves possible RNA secondary structures present in the RBS-2, embedded in the LP region, permitting translation of the downstream gene. (**e**) CRISPR-Cas Nuclease, Cas12a nuclease (grey shape) identifies the target sequence based on complementarity to gRNA (red line) and cleaves the complementary DNA strand, creating staggered ends. (**f**) CRISPRi, guided by a gRNA, an inactive version of Cas12a, dCas12a, targets a specific gene and obstructs the transcription by interrupting RNA polymerase activity either by binding to an exonic region (Elongation block) or the promoter region (Initiation block). (**g**) Riboswitch, the presence or absence of a ligand effector defines the occurrence of translation and, therefore, the ON/OFF gene expression switch. In the absence of the ligand, the ribosome binds the RBS and initiates translation (Translation ON). In the presence of the effector, it hybridizes with the RNA leading to a conformational change that renders the RBS inaccessible to the ribosome and impedes translation (Translation OFF). (**h**) A Ribo-regulator is composed of a trans-activating RNA (taRNA) and a cis-repressed RNA (crRNA). When expressed alone, the crRNA is present in a conformation that precludes the RBS from binding to the ribosome (taRNA OFF). The expression of a taRNA (taRNA ON), containing a region complementary to the 5′ of the crRNA (coloured in blue), binds to the crRNA, restricting it to a conformational change that makes the RBS accessible for ribosome binding. (**i**) Toehold switch, the toehold RNA structure, is located upstream of the translation start site (TSS, coloured in red) and the toehold hairpin structure renders the RBS inaccessible to the ribosome, precluding the initiation of the translation. A second RNA molecule (trigger RNA), containing a sequence complementary to the toehold 5′ sequence of the toehold RNA structure (coloured in blue), binds to and stretches the toehold structure and frees up the RBS, allowing translation initiation. (**j**) Small transcription activating RNAs (STARs), an RNA molecule (RNA-1) containing an anti-terminator sequence, hybridizes upstream of a target gene. The presence of the anti-terminator forces the RNA polymerase to end transcription. The presence of a second regulatory RNA (RNA-2) capable of binding to a complementary region of RNA-1 (coloured in blue) forces a conformational change of RNA-1, preventing hairpin formation and enabling transcription.

Dynamic gene expression, which allows decoupling growth and production, can be helpful in cases where the target product is toxic for the host. Such a dynamic regime can be achieved with promoters regulated by environmental conditions, such as oxygen, CO_2_ partial pressure and light intensity [156], or by chemical inducers that can either activate or repress expression (Figure 1a). The current cyanobacterial engineering toolbox provides several inducible promoters.

P*_trc_* is a popular inducible promoter for the metabolic engineering of *E. coli* derived from the lactose operon system (*lac* operon). The system is induced by isopropyl ß-D-1-thiogalactopyranoside (IPTG), a molecular mimic of the natural lactose inducer and repressed in its absence [157]. To modulate expression strength, synthetic variants were created by modification of the spacer sequence between the -35 and -10 boxes and tested both in Syn7942 and Syn6803 [150,158]. Albers et al. placed *lacI* (*lac* repressor) under the control of the housekeeping gene *sigA* encoding the Sigma factor A, ensuring strong constitutive expression of the repressor [159]. Combined with the P*_sca6–__2_* promoter, a modified version of P*_trc_*, they achieved a 10-fold induction ratio after adding IPTG [159].

In *E. coli*, the transcription factor NanR can recognize and bind to specific DNA sequences “GGTATA”, called nan box and located within the promoter region, downstream of the RNA polymerase binding site [160]. Thus, it can repress the downstream gene expression. N-acetylneuraminic acid (Neu5Ac), a common sugar moiety, can reduce the NanR affinity towards nan box sites and favour gene expression [160]. Sun et al. generated, in Syn2973 and Syn7942, a controllable switch sensing machinery for gene expression that responds to Neu5Ac inducer [161]. Expression of *lacZ* gene (encoding β-galactosidase) is driven by the synthetic promoter P*_J23119H10_*, containing two nan box sequences. Expression of *lacZ* is inhibited by the activity of NanR, whose synthesis is driven by P*_trc_*. Efficient dose-response expression of *lacZ* is achieved upon application of different concentrations of Neu5Ac.

The phage-derived TetR-regulated promoter P*_LtetO-1_*, inducible by anhydrotetracycline (aTc), was modified to obtain a variant applicable for Syn6803. Alterations of only a few base pairs upstream of the -10 consensus sequence resulted in variant P*_L03_*, which displayed a wide dynamic range in Syn6803 (290-fold induction upon aTc induction under red light) [162].

Metal native inducible promoters were also tested in Syn6803 but with limited success [163,164]. The nickel-inducible P*_nrsB_* efficiently promoted heterologous ethanol production upon Ni^2+^ addition, but cell viability defects compromised its use under high concentrations of Ni^2+^ [165]. The cobalt inducible promoter P*_coaT_* has successfully been employed for heterologous triterpenoid biosynthesis in Syn6803, but the need to work in Co^2+^ depleted media hampered cell growth [166].

Arabinose inducible promoters such as P*_BAD_* are the most effective inducible promoters in cyanobacteria and are functional in both Syn6803 and Syn7942 [167,168]. P*_BAD_* works in concert with the repressor AraC. In the absence of *L*-arabinose, the transcriptional regulator AraC is active and represses transcription from P*_BAD_*, while it is deactivated in the presence of *L*-arabinose, resulting in the induction of P*_BAD_* controlled gene expression. This system enables tight gene expression control in Syn7942. It was applied for photomixotrophic growth with arabinose utilization in the dark to improve bioproduction robustness under diurnal conditions [168].

Further examples of inducible promoters are the rhamnose RhaS-regulated promoter P*_rhaBAD_* [169] and P*_vanCC_*, originating from *Caulobacter crescentus* [170], both showing tight regulation in the absence of the inducer, a linear response to inducer titration and an excellent dynamic range in Syn7942 and Syn6803 and Syn6803, respectively.

However, for large-scale industrial bioprocesses, the use of external chemical inducers is unpreferred because it significantly adds to the production costs and poses environmental risks, for instance, due to the usage of heavy metals [153]. Promoters tunable by cheap, non-toxic compounds or environmental/physical signals may overcome this problem (Figure 1b).

#### 4.1.4. Environmentally Inducible Promoters

Several promoters activated by specific environmental inputs, such as light and oxygen, have been tested in cyanobacteria [156,171,172,173]. A common problem in strain engineering is the competition for precursors or cofactors required for production with native pathways. If the native pathway is essential for growth and cannot be deleted, this is addressed by downregulation of the competing activity [174]. This is achieved by exchanging native promoters with weaker ones or using promoters whose activity can be controlled by inducer or repressor titration [174].

In Syn6803, differential expression of the yellow fluorescent protein (YFP) was achieved by creating a dark inducible gene expression system based on EnvZ/OmpR two-component system of *E. coli* [175]. The dark inducible sensor (Cph8) is a chimeric protein composed of Syn6803’s phytochrome Cph1 and the histidine kinase domain EnvZ-OmpR. Dark conditions are sensed by Cph8 and lead to the dephosphorylation of EnvZ and phosphorylation of its corresponding regulator protein OmpR. The subsequent binding of the phosphorylated OmpR (OmpR-P) to the promoter *ompC* activates the promoter, prompting gene expression [175]. This tunable expression system represents an elegant strategy that can be exploited to produce light-sensitive proteins.

By contrast, Abe et al. exploited the cyanobacterial light-harvesting antenna, the phycobilisome, to build a light-sensing gene expression device composed of the green light receptor CcaS, the response sensor CcaR and the P*_cpcG2_* naturally controlling the phycobilisome linker gene *cpcG2* [176]. They regulated gene expression by shifting from red to green-light illumination and achieved expression levels comparable to P*_trc_* with an optimized system (Figure 1b) [171]. The same system was combined with the T7 phage RNA polymerase expression system, largely used in *E. coli* for heterologous protein expression. In this system, the P*_cpcG2_* controls the expression of T7 polymerase, which transcribes T7 promoter (P*_T7_*) controlled genes. Tight control of gene expression was achieved, and the expression level was close to that achieved with the super-strong promoter P*_cpc560_* [172]. T7 RNA polymerase-based transcriptional systems have also been successfully applied in Syn7002 [177], Syn7942 [178] and Syn2973 [139] for strong gene expression.

The fumarate and nitrate-reducing system (FNR), which acts as a global gene expression regulator in *E. coli* under anaerobic conditions [179], was adapted for Syn6803, creating an oxygen-responsive system based on FNR-activated promoter P*_FNR_* [173]. This new device was used to create a logic AND gate, a two-level tunable gene expression system, in Syn6803 by pairing it with a P*_tet_* promoter and the *Salmonella typhimurium* pathogenicity island 1 type III secretion system, which is required for virulence during intestinal infection [173]. It consists of promoter P*_sicA_* activated when the chaperone SicA and the transcriptional regulator InvF form a complex [180]. The transcription of *sicA* is boosted under anaerobic conditions, while InvF is under the control of P*_tet_* and only formed in the presence of aTc. In the presence of both physical and chemical inducers, SicA and InvF form a complex that actives P*_sicA_* and, therefore, the downstream target gene, with limited leakiness and conspicuous dynamic range [173].

Given the multiple stress conditions and parameters to be evaluated when designing an efficient photosynthetic microbial cell factory, physical and chemical inducible promoters would potentially keep gene expression in tune with physiological needs dictated by the environment. Although understudied, the development of these promoters and their characterization in different cyanobacteria species increases rapidly, showing great promise as tunable gene expression systems.

### 4.2. Designing Ribosomal Binding Sites for Improving Gene Expression

While promoters act on the transcription rate, the translation rate heavily depends on the ribosome binding site (RBS). Translation initiation is fundamental for controlling protein expression; therefore, the design of short sequences with elevated ribosome binding affinity enables a significant increase in translation initiation.

#### 4.2.1. Ribosome Binding Site (RBS)

The RBS, also known as Shine-Dalgarno (SD) sequence, is a relatively short sequence (around 6 bp) that is located at the 5′-untranslated region (5′-UTR) of the mRNA generally 7 to 11 bp upstream from the AUG start codon. The 16S ribosomal RNA (rRNA), part of the 30S small ribosomal subunit in prokaryotes, binds to the RBS by a complementary 3′ anti-SD sequence forming the primary complex for translation initiation. The SD/anti-SD complementarity, presence of secondary structures and the distance between RBS and the start codon can determine the overall RBS strength [181]. Despite a standard anti-SD sequence (CCUCC), the typical RBS of *E. coli*, 5′-GGAGG-3′, is less preferred in cyanobacteria. This sequence is only found in the UTR of 26% of all Syn6803 genes (compared to 57% in *E. coli*) and it is typically located 9–11 bp upstream of the start codon (7–9 bp in *E. coli*) [182]. This apparent difference in UTR structure suggests a limited reusability of standardized parts characterized in *E. coli* and profuse efforts were made to characterize alternative cyanobacterial RBSs. Nevertheless, Englund et al. found that a test set of 11 different RBSs resulted in similar relative translation rates in *E. coli* and Syn6803, in stark contrast to the results obtained from a study of the same group testing promoter function in these two species [152]. In the same strain, 20 endogenous RBSs were characterized by inserting the RBS sequences upstream of the strong promoter P*_trc1O_* [141]. RBS-*ndhJ* (Subunit J of the type 1 NADH dehydrogenase) and RBS-*psaF* (Subunit F of photosystem 1) accounted for the highest activities for translation initiation, while RBS-*cpcB*, RBS-*rbcL* and RBS-*psbA2* failed to promote translation. The discrepancy in translation efficiency in the native and engineered setting may be attributable to the genetic context [139], pointing to the importance of taking the up- and downstream regions into account when designing RBSs.

Several synthetic RBSs designed using the RBS calculator [183] have been characterized in cyanobacteria species, including Syn7002, Syn7942, Ana7120, Syn6803 and *Leptolyngbya* sp. PCC BL0902 (Lep0902), adding to the synthetic biology toolbox of these organisms [117,154]. The efficacy of rationally designed RBSs was then efficiently exploited for the heterologous ethylene production in Syn6803 [184,185].

One of the potential drawbacks that can affect gene expression is the formation of secondary RNA structures at the level of the 5′-UTR-GOI junction, hampering RBS accessibility for the 30S ribosome and therefore obstructing translation initiation [186]. Additionally, regulatory DNA sequences downstream of the transcription start site, contained in many synthetic promoters, are transcribed, resulting in the accidental incorporation of nucleotides at the 5′ untranslated regions of the transcript [187]. These insertions may alter RNA stability and reduce translation efficiency and predictability [188]. The use of genetic insulators to leapfrog these events is discussed below.

#### 4.2.2. RiboJ

RiboJ is formed by the sTRSV-ribozyme26 of the satellite tobacco ringspot virus RNA [189] and a 23-nucleotide synthetic hairpin [190]. When placed upstream of an RBS, sTRV cleaves the 5′-UTR of the mRNA [191]. This leads to a standardized 5′-UTR of the transcript that is “insulated” from the genetic context and provides more reliable expression (Figure 1c) and was also found to improve expression by improving ribosome access to the RBS [190,191]. This approach was successfully applied to engineer Syn6803 for 1-butanol production. A RiboJ insulator sequence was placed between the P*_trccore_* promoter and the genes *npht*7 and *phaB* (encoding for aceto-acetyl CoA synthase and aceto-acetyl-CoA reductase, respectively), leading to a 5.3-fold increase in 1-butanol production [192].

RiboJ cleavage was also tested in conjunction with a synthetic RBS ([A/G]AAGGAGGT, see above) and found to increase translation of the YFP reporter in Ana7120, Syn6803 and Lep0902 but not in Syn7942 [117]. The difference in RiboJ efficiency between different bacterial strains was explained by a single nucleotide polymorphism (SNP) near the catalytic RiboJ site [188].

#### 4.2.3. Bicistronic Design (BCD)

The BCD (Figure 1d) is characterized by a short leader peptide (LP) and two RBSs, one located upstream of the LP (RBS-1) and a second (RBS-2) embedded within the LP sequence, just upstream of the gene of interest (GOI). The helicase activity of the ribosome translating the LP sequence resolves possible secondary RNA structures present in the RBS-2 that could otherwise hinder translation initiation of the downstream GOI (Figure 1 d) [193]. Both RiboJ and BCD were alternatively used to increase translation efficiency of proteins of the methylerythritol 4-phosphate (MEP) pathway in Syn6803 [194], thus showing the applicability in metabolic pathway engineering. Furthermore, the BCD design was recently ported to Syn6803 to improve translation efficiency of a hydrogenase for heterologous hydrogen production [195], demonstrating the practicality of this simple synthetic tool for expressing complex proteins. This system has been so far used only in Syn6803 and more applications in different species will need to be set up to demonstrate its general efficacy in cyanobacteria.

### 4.3. Using CRISPR for Gene Expression Regulation and Markerless Genome Editing

Due to the cyanobacterial polyploid genome organization, genome editing requires extended segregation periods to reach homogeneous populations. The clustered regularly interspaced short palindromic repeats (CRISPR) and its associated protein Cas (CRISPR/Cas) facilitate the genomic mutation frequency and speed up the segregation process. The Cas nuclease, directed by a guide RNA to a specific locus downstream of a protospacer adjacent motif (PAM), can precisely create double-stranded DNA cleavage that can be repaired by homology-directed repair, thus easing precise genome editing (Figure 1e). CRISPR/Cas was first applied as a genome editing tool in 2012 [196] but only exploited in cyanobacteria in 2016 and then established for genome modification in several cyanobacterial species [18]. Wendt et al. coupled the CRISPR system with the nuclease Cas9 from *Streptococcus pyogenes* [57]. While they successfully deleted the *nblA* gene responsible for phycobilisome degradation, they also experienced a toxic effect of Cas9 when constitutively expressed in Syn2973. This can be circumnavigated by employing inducible expression or weaker promoters [57,197] or the expression of Cas12a from *Francisella novicida*. The expression of this enzyme was reported to exert much lesser toxicity and it was efficiently used for markerless knock-ins, knock-outs and point mutations in Syn6803, Syn2973 and Ana7120, with about 20% correct genome edits [47].

More recent work reported a base editing tool based on CRISPR-Cas and deamination, which was successfully employed to introduce premature stop codons in genes of the glycogen synthesis pathways in Syn7942, thereby enabling the redirection of carbon into target products [198].

For essential genes which cannot be deleted, CRISPR interference (CRISPRi) that utilizes an enzymatically inactive “dead” Cas protein (dCas) can enable the downregulation of gene expression (Figure 1f). Experiments with inducible expression of dCas9 showcased applicability as a tunable gene repression system [199]. Using a CRISPR system, particularly CRISPRi, offers a substantial advantage for dynamic regulation of biosynthetic pathways to redirect the carbon flux toward a targeted chemical. Lactate production was propelled in Syn7002 upon overexpressing the lactate dehydrogenase (LDH) of *Bacillus subtilis* combined with downregulation of the glutamine synthetase I gene *glnA* through CRISPRi, which resulted in decreased ammonium uptake and an augmented level of the LDH substrate pyruvate [200]. A modest increase in succinic acid titer was achieved in Syn7942 by silencing glucose-1-phosphate adenylyl transferase (*glgC*) responsible for glycogen synthesis and the knock-in of two enzymes of the tricarboxylic acid cycle (TCA), phosphoenolpyruvate carboxylase (*pepC*) and citrate synthase (*gltA*) [155]. More recent work targeted additional genes via CRISPRi, resulting in succinic acid production of almost 9 g/L [201].

One of the great advantages of CRISPRi is the possibility of including different gRNAs in a single expression cassette, simultaneously targeting several DNA loci. This multiplexing nature of CRISPRi was exploited to simultaneously repress six lipid biosynthesis pathway genes, increasing the fatty acid production in Syn6803 [202]. Recently, CRISPRi/Cas12a mediated multiple gene interference was used in Syn7942 to partition carbon assimilation toward the triterpenoid squalene [203]. Both examples demonstrate the applicability for the metabolic engineering of cyanobacteria.

The use of CRISPRi as a modular and flexible gene regulation system that responds to different stimuli was recently showcased by combining it with two physically and chemically inducible promoters [204]. The synthetic systems used the circadian clock as a blueprint for a genetic circuit that computes an NGATE logic. In Syn 7942, the circadian rhythm is controlled by the *kaiABC* operon, which encodes a posttranslational oscillator complex that responds to environmental stimuli [205]. It works together with a two-component system, in which SasA (*Synechococcus* adaptive sensor A) intercepts with the complex and transmits the information to the second component RpaA (regulator of phycobilisome association A), whose phosphorylation state influences the expression of circadian clock genes [205]. The designed genetic circuit was composed of YFP controlled by the IPTG-inducible promoter P*_trc_*, a sgRNA targeting P*_trc_*, driven by a dark-induced promoter P*_urf_* and a dCas9 controlled by the aTc-inducible promoter P*_tet_*. In the presence of IPTG, downregulation of YFP is only achieved in the presence of the two inputs: darkness and aTc addition; light conditions, instead, lead to the deactivation of P*_urf_* and derepression of YFP [204].

In 2020, multiple metabolic engineering targets, such as high-light and CO_2_ stress acclimation regulator genes, were identified in Syn6803 by creating the first cyanobacterial CRISPRi library comprising 10,498 clones [200]. Such libraries are a promising resource for identifying target genes relevant to biotechnological applications.

CRISPR-based techniques are particularly attractive for cyanobacterial strain engineering as no interspecies efficacy differences have been observed, in contrast to the other synthetic biology tools available for cyanobacteria. Overall, the use of Cas12a for generating markerless mutants may be better suited than Cas9. It does not exhibit toxicity issues; it requires only a single pre-crRNA array to target multiple genes and a 42 nucleotide RNA is sufficient and significantly shorter than the 100 nucleotides required for the Cas9 system, rendering all experiments cheaper. Moreover, the YTN (CTN or TTN) PAM sequence necessary for Cas12a is more often present than the canonical NGG PAM sequence proper of Cas9, opening the doors to more target sequences.

### 4.4. RNA-based Regulatory System

Post-transcriptional gene expression can be modulated by engineering mRNA stability and translation efficiency. This is often achieved by small regulatory RNAs (sRNAs) and a variety of synthetic variants of these RNA-based devices have been developed to aid metabolic engineering [206,207]. The use of RNA in synthetic biology confers several advantages in (a) terms of specificity since the cis-element can act on a specific RNA substrate; (b) programmability as the alteration of the RNA structure can be easily controlled; (c) orthogonality, because of their high degree of insularity that can prevent obstruction with other cellular processes; and (d) cell economy because of the lack of translation processes [207]. Some promising regulatory RNA elements applied in cyanobacteria include riboswitches (Figure 1g), riboregulators (Figure 1h), toehold switches (Figure 1i) and small transcription activating RNAs (STARs) (Figure 1j), are described in the following section.

#### 4.4.1. Riboswitches

Riboswitches are cis-acting RNA elements that operate at the 5′-UTR of mRNA [208,209]. They are composed of two partially overlapping domains, an aptamer domain recognized by an effector molecule, whose binding creates a conformational change of the RNA structure, and an expression platform domain that can modulate gene expression by altering the transcription termination or by repressing the translation initiation (Figure 1g) [210]. Riboswitches are widespread in both Eukaryotic and Prokaryotic organisms because of their implication in regulating many metabolic pathways [211]. The best-characterized cyanobacterial riboswitch is a theophylline-dependent riboswitch. A synthetic version of this riboswitch was developed for Syn7942 and led to a 190-fold induction of a luciferase reporter [212]. The same device was successfully adapted for altering the circadian clock in Syn7942 by putting *kaiC* expression (part of the *kaiABC* operon, see above) under control of the theophylline inducible riboswitch [212]. A set of six different synthetic theophylline riboswitches, previously tested in various bacterial species, were adapted for use in Syn7942, Lep0902, Ana7120 and *Synechocystis* sp. strain WHSyn [213]. All riboswitches showed a wide dynamic range of induction in these cyanobacterial species and tight expression of the fluorescent protein YFP with minimal leakage [213]. Another theophylline riboswitch was developed for Syn6803. Its characterization showed its ability to tightly control the expression of a green fluorescent protein (GFP) reporter and further disclosed the importance of the N-terminal sequence of the riboswitch, whose secondary structures can affect translation efficiency [214].

A tunable gene expression system has been engineered by coupling the aTc-induced P*_L03_* promoter (see above), the theophylline-riboswitch and CRISPR-dCas9, to control gene repression of *glnA*, a gene essential for nitrogen assimilation, in Ana7120 [48]. Using a *L03* promoter alone to drive the expression of dCas9 was insufficient to ensure tight repression of *glnA*. However, the insertion of theophylline-riboswitch between the P*_L03_* promoter and dCas9, in the presence of aTc and theophylline, robustly repressed *glnA* expression [48], demonstrating its utility for strictly controlling dCas9 expression for titratable regulation of genes.

Another elegant approach for controlling spatio-temporal gene induction was created in Ana7120 by coupling an adapted version of the *Bacillus subtilis* transcriptional ON riboswitch theo/pbuE* and pbuE/pbuE*, responding to theophylline or adenine respectively, with the heterocyst-specific promoter P*_nifB_* and a vegetative cell-specific promoter P*_rbcL_* [215]. Both riboswitches were able to achieve gene induction depending on cellular differentiation.

Two riboswitches endogenous to cyanobacteria have been tested for heterologous gene expression control with differing success. The cobalamin riboswitch, which regulates the production of vitamin B_12_ in *Synechococcus elongatus* 73,109 (Syn73109), was tested in Syn7002 and showed 6-fold induction of gene reporter expression [216]. In contrast, the glutamine riboswitch displayed poor binding affinity and consequentially low gene expression control [217].

Riboswitches have shown efficient and strict regulation of translation in several cyanobacterial strains. Given that they require only an aptamer-ligand interaction, they represent a simple and reliable synthetic biology tool for engineering cyanobacteria.

#### 4.4.2. Riboregulators

Other RNA cis-acting elements for independent control of gene expression are riboregulators. They are composed of a cis-repressed RNA (crRNA) and a trans-activating RNA (ta-RNA). The crRNA hybridizes with the RBS, thereby blocking ribosome binding. The taRNA contains a sequence complementary to the internal loop region of the crRNA; taRNA binds to crRNA, constraining its ability to hybridize to the RBS and alleviating translation inhibition of the target gene (Figure 1h). Abe et al. [218] were the first to report the application of a ribo-regulator in *Synechocystis*. They used a mutant of the *E. coli* crR12-taR12 ribo-regulator (crR*2/taR*2), in which crRNA and taRNA expression were controlled by the constitutive P*_trc_* and the Ni^2+^-inducible *nrsB* promoter, respectively. With this configuration, the circuit is constitutively in an OFF state and translation (ON state) is only induced upon the addition of Ni^2+^. In Syn6803 this system led to a 13-fold increment in the GFP signal [218,219].

Follow-up work improved the gene expression and structure stability by inserting a sequence for binding RNA chaperone Hfq (a protein that increases the stability of sRNAs by protecting them from endo-nucleolytic cleavage) and a rho-independent transcription terminator sequence. These modifications promote correct RNA hybridization, including accurate and efficient gene regulation ability [220].

Sun et al. generated a binary regulation of target gene (*lacZ*) by coupling the NanR/Neu5Ac biosensor system (see above) to a theophylline-inducible ribo-regulator [161]. The system is composed by three different expression cassettes encoding for the chaperonin Hfq, NanR and a riboregulatory synthetic RNA sequence (sRNA) complementary to the target gene, regulated by P*_trc_*, P*_cpc560_* and P*_J23119H10_*, respectively. Expression of *lacZ* is controlled by a theophylline inducible riboswitch (see above), while its expression level can be altered by sRNA regulatory element, which, in turn, is controlled by NanR/Neu5Ac switch. Tunable gene expression level was achieved depending on the concentrations of the two inducers, theophylline and Neu5Ac.

While applications of ribo-regulator technology in cyanobacteria are still scarcely investigated, significant improvements were accomplished, as demonstrated by the 78-fold increment in dynamic range using an optimized version of the taR*2/crR*2 [219]. Furthermore, the use of this system to repress the *cyAbrA2* encoding a global transcriptional regulator in Syn6803 allowed a tunable ON/OFF device for regulating glycogen production, highlighting the applicability of riboregulators as a metabolic engineering tool in cyanobacteria [221].

#### 4.4.3. Toehold Switches and Small Transcription Activating RNAs (STARs)

Other classes of riboregulators such as the Toehold switches and Small Transcription Activating RNAs (STARs) (Figure 1i,j) account only for a few applications in cyanobacteria so far. Toehold switches are composed of a hairpin loop containing an RBS and a start codon upstream of the coding sequence. The hairpin loop precludes the ribosome attachment to the RBS. When a trigger RNA binds to a complementary sequence upstream of the hairpin (Toehold sequence), the RNA unfolds, and translation is activated [222] (Figure 1i).

STARs are gene regulatory systems consisting of two components: a GOI and an upstream target RNA that, after transcription, folds into an intrinsic terminator that hampers the downstream gene transcription. The second component is an RNA that binds the target RNA, prohibiting hairpin formation and allowing gene transcription [223] (Figure 1j). In a study comparing STARs and Toehold switch performance in Ana7120 by placing the trans-element under the control of the aTc inducible *L03* promoter and with LacZ as reporter, STARs showed substantial leakage activity while the Toehold ensured tight gene expression control [48].

Riboregulators can find their applicability in the future as effective multiple gene knockdown technology. In contrast to CRISPR, they do not rely on the presence of a PAM sequence, which makes the design more flexible. Compared to riboswitches, they allow a more robust regulation of gene expression; the possibility to be controlled independently from the promoter of the target gene makes them a very reliable gene regulatory system to be further exploited in cyanobacteria.

## 5. Metabolic Engineering Approaches for Increasing Cyanobacterial Production

Metabolic engineering can be defined as a strategy for perturbing cellular metabolic networks to improve the production of selected molecules [224]. Thus, metabolic engineering is a key approach for developing microbial platforms for the biosynthesis of molecules with valuable industrial properties. The potential of generating industrial-relevant molecules using light and CO_2_ as energy and carbon sources makes cyanobacteria attractive agents for sustainable bioproduction and they have been widely engineered to produce a vast array of different chemicals [10]. Here, we describe several metabolic engineering approaches adopted in cyanobacteria for increasing the production of fatty acids, carbohydrates and terpenoids. Industrially, the alkyl monoesters of fatty acids represent the starting material for biodiesel [225]; sugars such as glycogen may be used as a feedstock for bioethanol production [226,227] and terpenoids have broad industrial applications, from medical and agricultural to flavor and the fragrance industry [228,229].

### 5.1. Fatty Acid Production Engineering

**Figure 2:**
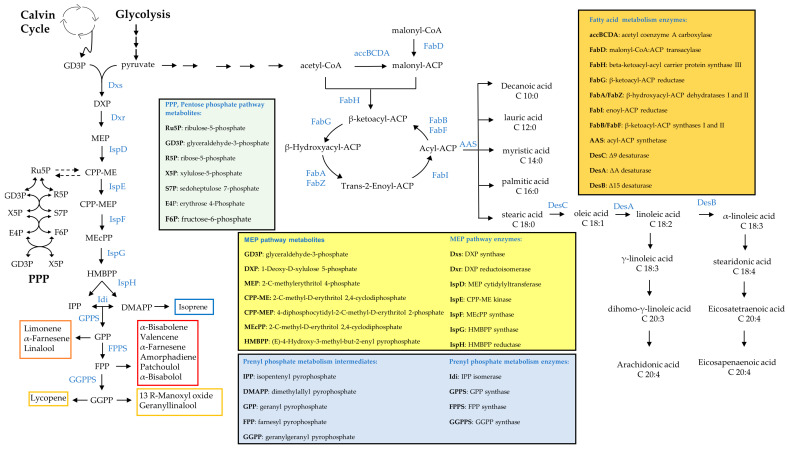
Schematic representation of biosynthetic routes for terpenoids and fatty acids.

Microbial fatty acid production aims to replace fossil fuel and environmentally destructive monocultures such as palm trees as sustainable sources of oleochemicals. However, microbial production needs to become more economically viable to be realized at scale. Here we review recent advances in metabolic engineering of fatty acid production in cyanobacteria. Fatty acid (FA) biosynthesis is highly conserved across prokaryotes and eukaryotes and highly regulated (Figure 2) [230]. Achieving a high fatty acid production in any microorganism requires its decoupling from growth either by metabolic engineering or environmental pressure as naturally achieved by oleaginous organisms, including some cyanobacteria [230,231]. Cyanobacteria present themselves as promising chassis for fatty acid production. Besides low feedstock requirements, one of the main advantages of cyanobacteria is their potential to secrete fatty acids into the media.

Liu et al. engineered Syn6803 to produce and secrete fatty acids to circumvent the biomass separation and extracting steps needed in other cases, producing a majority of 43% of C16:0 and a secretion yield of 197 mg L^−1^ of culture (Table 2) (Figure 2) [232]. They boosted the free fatty acid (FFA) level by sequentially introducing multiple copies of acyl-acyl carrier protein thio-esterase and acetyl-CoA carboxylase (ACC) genes, encoding rate-controlling enzymes in fatty acid synthesis (Figure 2). They also knocked out polyhydroxy-butyrate (PHB) synthesis to achieve the highest production levels (Table 2) [232]. In order to facilitate secretion, they also weakened the peptidoglycan layer by knocking out peptidoglycan assembly proteins [232]. However, a noticeable issue was that the cells became fragile and membrane damage occurred at low cell density [232]. Kato et al. were able to triple FFA secretion by using a two-phase culture, which alleviated lipid stress on the non-secreting strain dAS1T derived from Syn7942. Their system reached 0.64 mg L^−1^ FFA in the medium [233]. More recently, a non-model cyanobacterium, Syn11901, was found to produce an even higher titer of FFA [61], reaching 1.5 g L^−1^. Fatty acid alcohol synthesis is not naturally present in cyanobacteria but was engineered into Syn6803 by expressing a fatty acyl-CoA reductases (FAR) from mouse, jojoba and Arabidopsis, leading to a low initial product titer of around 10 µg L^−1^ [148]. Yao et al. picked another strategy and knocked out two enzymes involved in alkane synthesis in cyanobacteria and overexpressed a FAR from a marine bacterium, obtaining a slightly higher titer of 2.8 mg per gram cell dry weight (g CDW). The strain mainly produced hexadecanol and octa-decanol, but the availability of FAR with different chain-length specificities can be utilized to tailor the fatty alcohol profiles in cyanobacteria [234].

Santos-Merino et al. enabled alpha-linoleic acid (ALA), C18:3, production in Syn7942 via the introduction of two desaturases, deletion of *fadD* and overexpression of *fadF*, two genes involved in the fatty acid synthesis pathway of cyanobacteria (Figure 2), which enriched ALA to 22% of the total FAs pool. They also studied the role of each *fad* gene by deleting and overexpressing single genes and analyzing the fatty acid profile [235]. Thus, they demonstrated great versatility of the fatty acid metabolism in cyanobacteria and the possibility of making more complex fatty acids.

More recently, a higher amount of polyunsaturated fatty acids (PUFA) was produced in two engineered cyanobacteria strains: Lep0902, known to naturally produce a high amount of FA and Syn7002. Both strains produced ALA, but also stearidonic acid (SDA), C18:4 and eicosatetraenoic acid, C20:4, up to 40% of total FAs (Table 2 and Figure 2). To achieve this, Poole et al. overexpressed desaturase genes and a thylakoid membrane-promoting protein *vipp1*. The latter manipulation increases the formation of thylakoid membrane to which the desaturase reactions are localized [236].

Despite these advancements, maximal titers of various fatty acids are typically 10–50 times lower than those obtained with heterotrophs [230], a limitation that may be overcome with increasing knowledge on fatty acid synthesis in cyanobacteria [235]. The potential to tune the production of fatty acid-derived molecules in cyanobacteria, particularly for higher-value unsaturated PUFA with pharmaceutical applications, is promising [236].

### 5.2. Carbohydrate Production Engineering

Cyanobacteria naturally keep a high carbohydrate content as part of their osmotic regulatory system and accumulate them under stress conditions. Engineering high carbohydrate-producing strains has, therefore, the potential for enhancement via metabolic engineering. The primary use for high carbohydrate-producing cyanobacteria strains is as feedstock for growing industrially relevant microorganisms [56].

The cyanobacterium Syn7942 has been engineered to become a sucrose producer and exporter at rates competing with terrestrial equivalents such as sugar cane (Table 2). Relying on the natural production of sucrose, Ducat et al. overexpressed a sucrose permease gene *cscB* and knocked out invertase and an ADP-glucose pyro-phosphorylase [237] to reach a production of 36.1 mg L^−1^ h^−1^. The same phenotype was created in the UTEX 2973 strain (Table 1), which has a doubling time significantly superior to other cyanobacteria. Song et al. also turned Syn2973 into a high sucrose producer/exporter, reaching the same level as Syn7942 [55]. As Syn2973 is a significantly faster-growing strain and is more tolerant to abiotic stresses (Table 1), it could be a better choice to produce sucrose in an industrial setting [55]. Most recent work by Lin et al. doubled the sucrose production in Syn2973 by introducing the same *scsB* and additional overexpression of *sps* and *spp,* respectively, encoding for sucrose phosphate synthase and a sucrose phosphate phosphatase, reaching 79 mg L^−1^ h^−1^. They also managed to remove the need for high salt content in the media to induce sucrose production [238].

Besides sucrose, trehalose production is currently another attractive production target for cyanobacteria. Currently made by semi-enzymatic methods, it is an industrially relevant compound needed in the cosmetics industry but also useful for protection during organ transplant [239]. Recently Qiao et al. [239] have engineered a stress-tolerant strain of Syn7942 for trehalose production by blocking the sucrose production mechanism, introducing in place of *sps* a two-step pathway expressing a maltooligosyl trehalose synthase (MTS) and a maltooligosyl trehalose trehalo-hydrolase (MTH). They also expressed the insect trehalose transporter 1 TRET1 to secrete the sugar and engineered the glycogen metabolism to reach a titer of 5.7 g L^−1^ (Table 2).

Another exciting potential of cyanobacterial carbohydrates relies on valorizing their natural extracellular polymeric substances (EPS) as ingredients for the cosmetics and food industry as adjuvants and texture modifiers. With that aim in mind, Arias and Uggetti fully characterized a polysaccharide produced by *Cyanothece* sp. CCY 0110, named ‘cyanoflan’ (a biomaterial with potential use for wound healing) [240,241] and achieved 1.8 g L^−1^ of secreted EPS (Table 2).

Although not inherently highly valuable, cyanobacterial carbohydrate production has clear potential for various industrial applications, both for biofuel production and as a cosmetic ingredient and pharmaceutical additive production.

**Table 2:** Compound titers and relative cyanobacterial strains.

Compounds	Organism	Titer	Reference
Free Fatty acids (secreted)	Syn6803 Syn7942	197 mg L^−1^ 0.64 g L^−1^	[232] [233]
Free Fatty acids	Syn11901	1.5 g L^−1^	[61]
Fatty alcohol	Syn6803 Syn6803	9.7 μg OD_730_^−1^ L^−1^ 2.8 mg g_CDW_^−1^	[148] [234]
Alpha linoleic acid (C18:3)	Syn7942	22% of total FA	[235]
Alpha linoleic acid (C18:3), Stearidonic acid (C18:4), Eicosatetraenoic acid (C20:4)	Lep0902, Syn7002	40% of total FA	[236]
Total fatty acids	Syn7942 + *R. glutinis*	39 mg L^−1^	[242]
Sucrose	Syn7942 Syn2973 Syn2973	36.1 mg L^−1^ h^−1^ 35.5 mg L^−1^ h^−1^ 79 mg L^−1^ h^−1^	[237] [55] [238]
Trehalose	Syn7942	5.7 g L^−1^	[239]
Extracellular polysaccharide ‘cyanoflan’	*Cyanothece* sp. CCY 0110	1.8 g L^−1^	[240]
Isoprene (C_5_)	Syn7942 Syn6803	1.26 g L^−1^ 12.3 mg g_CDW_^−1^	[30] [243]
Limonene (C_10_)	Syn2973 Syn6803 Ana7120 Syn7002	16.4 mg L^−1^ 6.7 mg L^−1^ 3.6 μg L^−1^ 4 mg L^−1^	[244] [245] [246] [247]
(S)-linalool	Syn6803	11.4 mg L^−1^	[248]
β-phellandrene	Syn6803	10 mg g_CDW_^−1^	[249]
(E)-α-bisabolene (C_15_)	Syn6803 Syn7002	180 mg L^−1^ 0.6 mg L^−1^	[250] [247]
α-Farnesene	Ana7120 Syn7942	305.4 μg L^−1^ 12.99 mg L^−1^	[251] [252]
Amorphadiene	Syn7942	19.8 mg L^−1^	[253]
Valencene	Syn6803	9.6 mg L^−1^	[248]
β-Caryophyllene	Syn6803	N.R.*	[254]
Patchoulol	Syn6803	17.3 mg L^−1^	[255]
α-bisabolol	Syn6803	96.3 mg L^−1^	[255]
13R-manoyl oxide (C_20_)	Syn6803	2 mg L^−1^	[256]
Geranyllinalool (Floating and intracellular)	Syn6803	390 μg g_CDW_^−1^	[257]
Squalene (C_30_)	Syn7942 Syn6803	9.5 mg L^−1^ 5.1 mg L^−1^	[258] [259]
Lycopene (C_40_)	Syn6803	1.5 mg g_CDW_^−1^	[260]

### 5.3. Terpenoid Production Engineering

Unicellular, photosynthetic organisms are in the spotlight of microbial terpenoid production because these organisms possess traits that might benefit terpenoid production. Cyanobacteria synthesize terpenoids via the MEP pathway, of which a complete set of homologous genes has been identified. The flux through the terpenoid synthesis pathways is assumed to be higher, as in heterotrophs, because of the demand for chlorophyll and carotenoids to sustain photosynthesis and combat oxidative stress. Microalgae deliver the reducing equivalents strongly demanded by terpenoid production via photosynthesis while having a simpler cellular architecture and being more straightforwardly genetically manipulated than higher plants [228]. Despite these promises, terpenoid titers in engineered cyanobacteria or microalgae reported to date are very low, most often in the lower single-digit per mL range (Table 2). We here focus on some outstanding examples of terpene production from cyanobacteria.

Gao et al. [30] produced above 1 g L^−1^ of isoprene within three weeks in Syn7942 through upregulation of the native methylerythritol 4-phosphate (MEP) pathway (Figure 2). The key limiting steps identified in this study were 1-deoxy-D-xylulose 5-phosphate synthase (Dxs), 4-hydroxy-3-methylbut-2-enyl-diphosphate synthase (IspG) and isopentenyl pyrophosphate isomerase (Idi) (Figure 2), which were relieved by the integration of a second copy of the native *dxs* and *ispG* gene and a codon-optimized *idi* gene from *S. cerevisiae* under control of the strong, inducible P*_trc_* promoter. While *dxs* and *ispG* overexpression targeted kinetic bottlenecks of the MEP pathway (Figure 2), the heterologous isopentenyl isomerase was expressed to shift the ratio of the two hemiterpenes IPP and DMAPP towards the isoprene precursor DMAPP (Figure 2). Quantification of IPP and DMAPP, starting molecules of the prenyl phosphate metabolism (Figure 2), showed that this strategy successfully increased the DMAPP/IPP ratio by 130-fold. An interesting observation in this study was that the high specific isoprene production did not decrease the biomass yield but was balanced by an increased carbon fixation rate [30].

Following these results, Englund et al. [194] observed increased isoprene production in Syn6803 upon overexpression of either *dxs* or *fni* (encoding the native isopentenyl pyrophosphate isomerase). However, single overexpression of *ispG* did not result in higher isoprene production. Likely, the HMBPP synthase activity is not the primary limiting step of the *Synechococcus* MEP pathway and this strategy only becomes effective when other flux-constraining enzyme activities are upregulated [194]. Production of the sesquiterpenoids farnesene and amorphadiene and the triterpene squalene was likewise improved by lifting the metabolic bottlenecks given by Dxs and IPP isomerase activity (Figure 2) [251,253,261].

Chaves and Melis addressed low productivity by fusing the isoprene synthase characterized by low catalytic efficiency to the β-subunit of the highly expressed photosynthesis pigment phycocyanin encoded by *cpcB*. In the engineered strain, the abundance of the synthase was significantly increased and, in combination with the expression of a heterologous isopentenyl isomerase, resulted in a 62-fold improvement of the isoprene titer [243]. The group also successfully employed the same fusion strategy to improve a β-phellandrene synthase activity to produce this monoterpenoid [249].

Besides the canonical MEP Pathway, cyanobacteria are assumed to operate a shunt that channels intermediates of the pentose phosphate pathway into the lower MEP pathway under phototrophic conditions (Figure 2). This hypothesis is supported by the insensitivity of the MEP pathway in this organism to MEP pathway precursors and intermediates and its stimulation by some phosphorylated sugars [262,263].

This shunt might fuel the MEP pathway with intermediates from the pentose phosphate pathway (PPP), thereby bypassing the rate-limiting Dxs step and may have contributed to the increased limonene synthesis observed in Lin et al. [245]. The authors used a genome-scale metabolic model of Syn6803 to simulate the effect of the hypothetical connection between the Calvin-Benson-Cycle/PPP and the lower MEP pathway (Figure 2). The model extended with this shunt predicted that increased flux through this shortcut could increase limonene production. The model prediction was verified in vivo by overexpression of the PPP genes *rpi* and *rpe*, which pushed the limonene titers to more than 6 mg L^−1^ when combined with the simultaneous upregulation of geranyl pyrophosphate synthase (GPPS) activity. It remains to be elucidated if this increase is caused by a higher flux through the proposed PPP-MEP shunt (Figure 2).

As described above for *E. coli*, the recombinant expression of a mevalonate pathway likewise resulted in increased terpenoid production [264].

### 5.4. Nanocompartments Engineering

Bacterial nano-compartments (BNC) are intracellular structures composed of a selective proteinaceous scaffold that enclose a segment of a metabolic pathway [265]. Similarly to eukaryotic organelles, the external structure shields the internal enzymatic core from the cytosolic environment [265]. These structures may be exploited as cargo carriers for the delivery of molecules, as bioactive compounds with pharmacological properties and as agrochemicals and biomolecules within the scope of phyto-nanotechnology [266,267].

Exploring and gaining knowledge about cyanobacterial nano-compartments and their roles, is an interesting avenue for enhancing stress resistance and engineering novel metabolic pathways in nano-compartments.

One of the most commonly studied cyanobacterial nano-compartments are carboxysomes. Caroboxysomes were discovered in cyanobacteria in 1956 by Drew and Niklowitz; these protein compartments concentrate the CO_2_ to obtain the highest possible activity of the RuBisCO enzyme, which is essential for carbon fixation [268]. Plant scientists have obviously raised intense interest in this compartment and the engineering of agricultural plants for increasing plant carbon fixation efficiency with cyanobacterial carboxysomes has been attempted [269]. Delivery of enzymes or metabolic pathways into plant systems would ensure the transient expression of biomolecules, thus avoiding complicated transgene integration processes and the transfer of modified traits to the subsequent generations [266,267].

Stable integration of carboxysomes genes in the chloroplast is notoriously quite challenging [270]. Fang et al. report the first heterologous expression of Syn7942 β-carboxysome in *E. coli* using 19 genes split onto two synthetic plasmids [271]. Carboxysomes have an essential role in carbon fixation; therefore, gaining a better understanding of their assembly and function is essential in order one day to perhaps use them as a target to optimize cyanobacterial growth and to be able to eventually engineer better carbon fixation [272].

Besides carboxysomes, other nano-compartments exist in prokaryotic organisms, such as encapsulins [273]. Many different types of encapsulins have been discovered and genome mining studies were able to discover the presence of potentially dozens of novel families [274] involved in various domains, redox stress and iron mineralization, amongst others. Nichols et al. have investigated a nano-compartment encapsulating a cysteine desulfurase enzyme in Syn7942, which is upregulated upon sulfate starvation in cyanobacteria [275]. Besides their potential role in stress resistance, encapsulins could be engineered to encapsulate any enzyme or enzymatic pathway of interest. Nano-compartments can offer protease protection and isolate unstable intermediates in reaction mechanisms. Cyanobacterial encapsulins clearly represent an untapped and understudied area that could be promising for engineering more robust cyanobacteria and improving the efficiency of metabolic engineering efforts.

### 5.5. An Ecosystem Approach for a More Sustainable Production

The direct use of cyanobacteria as feedstock involves mixing the cells with other microorganisms, allowing them to either feed on the biomass and/or ferment the carbohydrates. Another strategy is integrating both cultures into a synthetic ecosystem to achieve production of the final compound of interest. Ideally the organisms of such a consortium would interact with and be mutually dependent on each other. Such a symbiotic culture could, for instance, be realized using cyanobacteria as the photosynthetic producer of carbon food sources used by an established industrial biotechnology workhorse such as *S. cerevisiae*.

Recent work has explored the use of cyanobacteria in fully characterized synthetic consortia of microorganisms symbiotically interacting to produce various compounds of interest, including the biopolymer polyhydroxy-butyrate (PHB) alpha-amylase [276] and has highlighted the challenges in establishing such systems. Hays et al. combined Syn7942, engineered for high sucrose production and export [237] with *Bacillus*, *E. coli* and *Saccharomyces* strains. The stability of each consortium over extended periods was assessed, as well as their response to various stresses and, finally, their ability to produce alpha-amylase and PHB. Challenges encountered were that heterotrophs needed a higher amount of sucrose than what was produced by the cyanobacteria to sustain growth after 24 h and the fact that a high optical density (OD) of cyanobacteria led to inhibition of the heterotrophs independently from the level of sucrose [253]. Potential partnering with a third microorganism might help to mitigate oxygen-deleterious side effects on the heterotroph. An interesting finding was that co-culturing cyanobacteria with other heterotrophs seemed to stimulate their growth [276], which could be related to oxygen removal that can be toxic for cyanobacteria at increased levels.

In another consortium involving a sucrose-producing cyanobacterium and three yeast strains, a similar issue of growth inhibition due to the presence of reactive oxygen species (ROS) was observed and alleviated by using a catalase enzyme [242]. The co-culture of Syn7942 (engineered to overexpress the proton/sucrose symporter gene, *cscB*, of *E. coli*) and the carotenogenic yeast *Rhodotorula glutinis* was able to achieve a higher rate of linoleic acid and palmitoleic acids compared to the yeast alone. Similarly, a positive impact of the heterotroph on the cyanobacteria growth was noticed [242].

Other systems have been studied involving a semi-controlled ecosystem growing cyanobacteria on wastewater, leading to high production of the biopolymer poly-hydroxy-alkanoate (PHA), a natural product made by some cyanobacteria when under stress, reviewed by Arias and Uggetti [277].

Although this consortium approach appears as a seducing alternative offering a sustainable and integrated system, harnessing various microorganisms’ strengths and combining them needs careful optimization. Optimizing such complex systems will probably require advanced metabolic engineering to design perfect partnerships. The potential for a photoproduction system involving cyanobacteria associated with one or multiple heterotroph microorganisms is vast and ecosystem engineering could allow tuning and controlling metabolite production at another level. Nevertheless, studies exploring the potential of these ecosystem approaches highlighted that, rather than focusing purely on metabolic engineering, product yield can also significantly be optimized by intentionally applying stress or other external factors; sometimes aiming for slowing growth purposefully or tuning down the expression to balance metabolic activities can lead to the best production.

## 6. Conclusions

This review covered the current state of cyanobacterial biotechnology, including cyanobacteria with industrially relevant traits, the synthetic biology tools available to control gene expression and a description of several metabolic engineering approaches applied for photosynthetic bioproduction.

We anticipate that more synthetic biology tools for the efficient development of cyanobacterial cell factories will be rapidly developed. Combined with large-scale efforts to generate genetic libraries (overexpression and deletion mutants), such as those available for *E. coli*, to improve understanding of cyanobacterial metabolism and physiology and to develop advanced photobioreactors, these capabilities will enable the full potential of cyanobacteria and sustainable and photosynthetic bioproduction to be realized.
